# SARS-CoV-2: Immune Response Elicited by Infection and Development of Vaccines and Treatments

**DOI:** 10.3389/fimmu.2020.569760

**Published:** 2020-12-11

**Authors:** Gisela Canedo-Marroquín, Farides Saavedra, Catalina A. Andrade, Roslye V. Berrios, Linmar Rodríguez-Guilarte, María C. Opazo, Claudia A. Riedel, Alexis M. Kalergis

**Affiliations:** ^1^ Millennium Institute of Immunology and Immunotherapy, Departamento de Genética Molecular y Microbiología, Facultad de Ciencias Biológicas, Pontificia Universidad Católica de Chile, Santiago, Chile; ^2^ Millennium Institute on Immunology and Immunotherapy Departamento de Ciencias Biológicas, Facultad de Ciencias de la Vida, Universidad Andrés Bello, Santiago, Chile; ^3^ Departamento de Endocrinología, Facultad de Medicina, Pontificia Universidad Católica de Chile, Santiago, Chile

**Keywords:** SARS-CoV-2, COVID-19, immune response, treatments, vaccines

## Abstract

The World Health Organization (WHO) announced in March a pandemic caused by Severe Acute Respiratory Syndrome Coronavirus 2 (SARS-CoV-2). This new infectious disease was named Coronavirus Disease 19 (COVID-19), and at October 2020, more than 39,000,000 cases of SARS-CoV-2 have been detected worldwide leading to near 1,100,000 deaths. Clinically, COVID-19 is characterized by clinical manifestations, such as fever, dry cough, headache, and in more severe cases, respiratory distress. Moreover, neurological-, cardiac-, and renal-related symptoms have also been described. Clinical evidence suggests that migration of immune cells to the affected organs can produce an exacerbated release of proinflammatory mediators that contribute to disease and render the immune response as a major player during the development of the COVID-19 disease. Due to the current sanitary situation, the development of vaccines is imperative. Up to the date, 42 prototypes are being tested in humans in different clinical stages, with 10 vaccine candidates undergoing evaluation in phase III clinical trials. In the same way, the search for an effective treatment to approach the most severe cases is also in constant advancement. Several potential therapies have been tested since COVID-19 was described, including antivirals, antiparasitic and immune modulators. Recently, clinical trials with hydroxychloroquine—a promising drug in the beginning—were suspended. In addition, the Food and Drug Administration (FDA) approved convalescent serum administration as a treatment for SARS-CoV-2 patients. Moreover, monoclonal antibody therapy is also under development to neutralize the virus and prevent infection. In this article, we describe the clinical manifestations and the immunological information available about COVID-19 disease. Furthermore, we discuss current therapies under study and the development of vaccines to prevent this disease.

## Introduction

Severe Acute Respiratory Syndrome Coronavirus 2 (SARS-CoV-2), denominated by the International Committee on Taxonomy of Viruses (ICTV) (1), was first isolated during an outbreak in Wuhan, the Chinese province of Hubei in December 2019. This virus belongs to *Coronaviridae* family and *betacoronavirus* subfamily, known to infect mammals, such as bats, mice, and pangolins. An example of this subfamily is Severe Acute Respiratory Syndrome Coronavirus (SARS-CoV), which caused an epidemic in 2002 involving 26 countries with over 8,000 cases (1–4).

Since the outbreak in Wuhan in December 2019, SARS-CoV-2 has demonstrated an accelerated contagious and spreading behavior (5). The fast transmission and the high number of cases affecting worldwide have made the management of virus spreading extremely difficult. The transmission of the virus is person-to-person through fomites and respiratory droplets (5, 6). Furthermore, fecal shedding has been shown up to 5 weeks after the clinical recovery (7–9). Therefore, it is hypothesized that fecal-oral transmission could be another propagation route for SARS-CoV-2 (10), with an incubation period that can last approximately up to 7 days after exposure to the virus (6, 11, 12). Interestingly, asymptomatic individuals display viral loads that have shown to be challenging to detect during the period of incubation (13, 14). Consequently, the spreading of the virus has no contention, and therefore researchers actively work to find vaccines and treatments for this pathogen.

In this article we discuss the current knowledge about the innate and adaptive immune response during coronavirus disease (COVID-19). Furthermore, we describe the scientific strategies currently undergoing testing for prophylaxis or treatment for COVID-19.

## SARS-CoV-2 Virion Characteristics and Target Receptor in Cells

**Figure 1:**
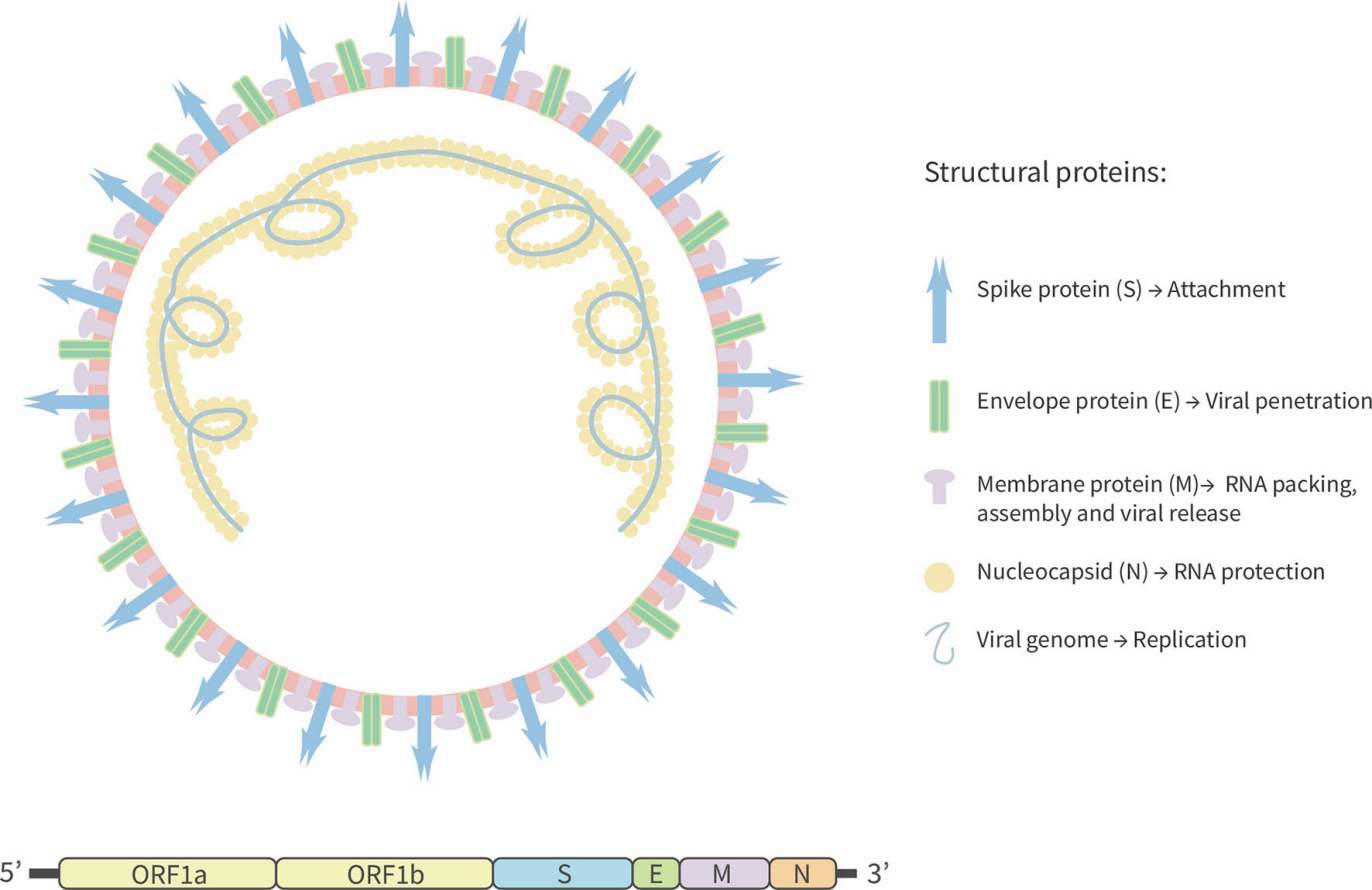
Schematic representation of SARS-CoV-2. SARS-CoV-2 is a positive-sense single-strand RNA enveloped virus. Viral genome encodes four structural proteins: Spike glycoprotein (S), envelope (E), Membrane (M), and Nucleocapsid (N) protein. Others 13 non-structural proteins are encoding by ORF segment 1ab.

SARS-CoV-2 is a positive-stranded RNA virus with an estimated genome size equal to 29.9 kb (15). In contrast, the genome size of previous pathogenic coronaviruses, such as SARS-CoV and the Middle East Respiratory Syndrome virus (MERS) is 27.9 kb, and 30.1 kb, respectively (3, 16). It has been predicted that SARS-CoV-2 has fourteen open reading frames (ORFs) that encode for four structural proteins: spike (S) that promotes the viral entry to host cell, membrane protein (M) that induces the membrane curvature and allows the union with nucleocapsid (N) protein. Additionally, the M protein interacts with the envelope protein (E) and allows virus assembly and release (15, 17, 18). Fifteen non-structural proteins are also encoded by the ORFab portion (15) (**Figure 1**). Similar to SARS-CoV, SARS-CoV-2 S-glycoprotein is cleaved by a transmembrane serine-protease 2 (TMPRSS2), producing two surface proteins S1 and S2 (19). The virus attaches to the host cell by the S1-domain by means of the receptor-binding domain (RBD), which binds to the Angiotensin Converted Enzyme 2 (ACE2) receptor to promote the viral fusion and the release of the viral genome into the host cells that is required for the production of new virions (20).

The ACE2 receptor can be expressed by cells from the respiratory system, arteries, heart, and digestive tract (20–22). In the respiratory tract, the receptor is expressed by pneumocytes type I and II located in the throat and lungs, as well as by alveolar macrophages (20). Despite the low presence of ACE2 both in the upper and lower respiratory tracts, the tropism for type II alveolar cells can be explained by the presence of proteases that contribute to the viral proteolytic processing required for SARS-CoV-2 entry (23). Three proteases have been described that can prime the S-protein: TMPRSS2, furin, and cathepsin B/L (22, 24, 25). TMPRSS2, as described above, cleaves the S protein in the S2 subunit at the RRAR motif, particularly in a highly basic domain (20). The furin protease also contains the cleavage RRAR sequence (23, 26). It has been described that furin can be secreted by the alveolar epithelium cells and work on neighboring cells (26). The synergy between furin and TMPRSS2 allows the viral entry to the cells (20, 27). According a predicted analysis cathepsin B/L are endosomal cysteine proteases that facilitate the entry of SARS-CoV into the cells; however, the contribution of these enzymes to SARS-CoV-2 entry has not been described (28).

## Clinical Features of SARS-CoV-2

**Figure 2:**
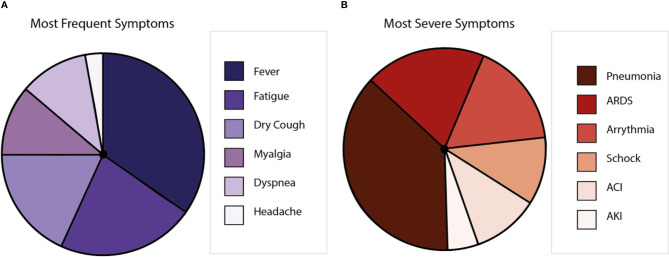
Symptoms caused by COVID-19 disease. **(A)** Is a representation of the most frequent symptoms in patients with SARS-CoV-2, where the most frequent symptom is the fever, followed by fatigue, dry cough, myalgia, dyspnea, and headache. **(B)** Is a representation of the most severe symptoms in patients with SARS-CoV-2, where pneumonia is the most common, followed by acute respiratory distress syndrome (ARDS), arrhythmia, shock, acute cardiac injury (ACI), and acute kidney injury (AKI).

COVID-19 displays clinical manifestations any time from 6 to 14 days after the exposure to SARS-CoV-2 (29). The most frequent symptoms are fever, nasal congestion, myalgia, headache, and cough (5, 30). However, anosmia and encephalitis have also been described for COVID-19 patients (31). Further, respiratory impairment occurs with severe clinical manifestations, such as respiratory distress and pneumonia (32). Comorbidities, including hypertension, diabetes, cardiovascular, and respiratory diseases, are closely associated with the severity of disease, mainly because patients with these ailments could develop pneumonia and require intensive care unit hospitalization, mechanical ventilation, and eventually extracorporeal membrane oxygenation (33). Symptoms could lead to multiple organ inflammation, resulting in organ failure (34, 35) that can cause death to the affected patient (30, 36) (**Figure 2**). Globally, the lethality is near to 2%–3% (37, 38), with the elderly population as the most susceptible (39). Interestingly, it has been reported that after the illness, a minor percentage of patients can show the reactivation of SARS-CoV-2 (40). Even more, virus reactivation can lead to the relapse of the patient (41, 42). So far, the hospitalization of the patients that relapse has not been described (43).

In addition to the involvement of lungs, heart, kidney, liver, and bowels, SARS-CoV-2 seems to reach the central nervous system (CNS), causing a broad range of clinical manifestations that will be discussed next (44). Similarly to other respiratory viruses (45–49), the ability of coronavirus to enter the CNS has been demonstrated by using murine animal models (50) and *in vitro* neuronal cell cultures (51, 52). Neurological symptoms were also shown for other coronaviruses, such as SARS-CoV and HCoV-OC43 (53–55). It has been suggested that neurons are a potential SARS-CoV-2 target, because these cells express the ACE2 receptor (31). Other cells from the CNS like astrocytes and oligodendrocytes are also targets of SARS-CoV-2 infection due to they also express the ACE2 receptor (56). In addition, a recent report showed that SARS-CoV-2 could be detected in neurons and microglia from deer mice at day 6 post-infection (44). Consistent with this notion, is the observation that 36.4% of patients can display several neurologic symptoms, including headache, encephalitis, impaired consciousness, and even the Guillain-Barré syndrome (57–59). Possible routes for the virus to enter into the CNS are the peripheral nerves and the blood-brain barrier (BBB), whether the virus uses immune cells or to go through the BBB remains to be defined (45, 60). Along these lines, it has been observed that patients infected by SARS-CoV-2 suffer from olfactory and taste disorders (OTDs) (57). Symptoms that seems to be more frequent in women than in men (61). Among the symptoms of taste disorders, dysgeusia and ageusia were observed, while hyposmia, and anosmia also occurred as olfactory disorders (62). It has been demonstrated that SARS-CoV can enter the CNS through the olfactory bulb (62). Since SARS-CoV-2 generates symptoms involving OTDs, it can be suggested that this virus can arrive at the olfactory bulb and enter the CNS (63). However, because the olfactory sensory neurons were found not to express the ACE2, SARS-CoV-2 could not infect them, unless it uses another receptor that has not been described yet (63).

Another coronavirus as HCoV-OC43, is thought to enter the CNS *via* the hematogenous route as an immune cell carry-on (64). It was suggested that SARS-CoV-2 also can enter the CNS through a hematogenous route. Using *in vitro* models for the BBB, the presence of ACE2 receptors was shown in blood vessels from the frontal cortex along with the brain microvascular endothelium (BMVEC) (65). Furthermore, the S protein seems capable of disrupting the BBB (65). Additionally, there is evidence for the presence of the virus in the cerebrospinal fluid (CSF), proving that SARS-CoV-2 can reach the CNS (62). An alternative to the hematogenous route for SARS-CoV-2 may be transporter or pass through the tight junctions from the epithelial cells in the choroid plexus and the endothelial cells from the veins located in the subarachnoid space, which are the cells that are part of the blood-CSF barrier (BCSFB) (65, 66).

Interestingly, the ability of SARS-CoV-2 to invade the CNS is thought to be associated with respiratory failure in patients with COVID-19, though this notion still remains to be conclusively defined (67, 68). Further studies are needed to evaluate the effect that the virus has on the CNS and the neurological symptoms that this pathogen causes in humans.

## The Immune Response to SARS-CoV-2

Although the immunopathology of coronaviruses remains poorly understood, the elucidation of the molecular and cellular mechanisms behind the immune response triggered by SARS-CoV-2 will help to develop vaccines and therapeutic strategies to control the infection or to improve the clinical progression of patients. Because SARS-CoV-2 is a novel virus, the immune response elicited by this pathogen is not yet comprehended. In this section, we discuss the main findings relative to the immune response to the virus and how this response affects the lungs.

### The Innate Immune Response Induced by SARS-CoV-2

Much remains to be understood about the molecular immune mechanism involved in SARS-COV-2 infection. Commonly, pathogen-associated molecular patterns (PAMPs) from microorganisms are recognized by pattern-recognition receptors (PRRs) (69). When viral fusion occurs, the viral genome can be recognized by various PRRs expressed by host cells (70–72). Among the PRRs that recognize viral RNA, it is essential to highlight toll-like receptors (TLR) 7 and 8 that trigger the myeloid differentiation primary response (MyD88) pathway upon binding to viral ssRNA (73, 74). Additionally, viral proteins can be recognized by TLRs and trigger the TLR4-MyD88 pathway (75). All these pathways promote the expression of type I IFNs (73, 76). Nevertheless, a deficient type I IFN response is observed during SARS-CoV-2 infection *in vitro* (77). An inefficient type I and type III IFN response has been suggested to associated with increased patient fatality. No IFN-β and IFN-λ could be detected in plasma samples and lung biopsies from SARS-CoV-2 individuals (78, 79). Type I interferon levels increased in patients suffering from severe disease and that improved after a critical condition (78). Even though the TLR-4 pathway involves the activation of the Tank-binding kinase 1 (TBK1), studies have shown that this kinase does not get activated during an *in vitro* infection with SARS-CoV-2 (77). All these *in vitro* results suggest that SARS-CoV-2 could inhibit the IFN pathway at one or more of the steps mentioned above. Contrary to the *in vitro* results, during infection of mice with SARS-CoV-2, an increase in IFN-α and TBK-1 was observed, with a peak at day 6 post-challenge, followed by a decrease in the expression of these molecules (44). These data suggest that there might be an IFN response during the infection *in vivo*, which declines later as the viral loads from the lung tissue (44).

Hematological studies have shown a progressive increase in the number of neutrophils in the peripheral blood of COVID-19 patients (40, 80–82), especially in those cases with respiratory distress (83). Furthermore, pulmonary infiltration has been found in autopsies of COVID-19 patients (84). On the other hand, COVID-19 patients had shown low eosinophil levels that could be considered as a laboratory biomarker (40, 80, 81, 85). Moreover, the presence of natural killer (NK) cells is reduced in COVID-19 patients, which might be associated with higher levels of chemokines ligand (CXCL) 9 and 16 (77). It is thought that a decrease in IFN-γ secretion could be associated with an impairment of the antiviral immune response (81, 86–89).

Some patients have shown a cytokine storm release (CRS) response associated with a negative prognosis, including death (90). CRS is an excessive inflammatory response induced by the SARS-CoV-2 infection, which in severe cases consists of the reduction of T cells and diffusing airway damage due to the infiltration of immune cells and the hyaline membrane formation (91, 92). COVID-19 severe cases show increased levels of several cytokines and chemokines, at least five-fold or more than healthy controls (93). Among them IL-2, IL-7, granulocyte-colony stimulating factor (G-SCF), interferon gamma-induced protein 10 (IP-10), monocyte chemoattractant protein-1 (MCP-1), macrophage inflammatory protein 1A (MIP-1A), monocyte chemotactic protein-3 (MCP-3), tumor necrosis factor alpha (TNF-α), IL-6, and IL-1RA had been shown to associate with the pro-inflammatory profile (5, 90, 93–97). Moreover, IL-6 levels have been directly corelated to viral loads (95). Also, increased IL-6 levels are detected before the intubation for mechanical ventilation (98, 99). IL-6 levels are linked to elevated IP-10, MCP-3, and IL-1RA levels and are associated with fatal outcomes (95, 100).

**Figure 3:**
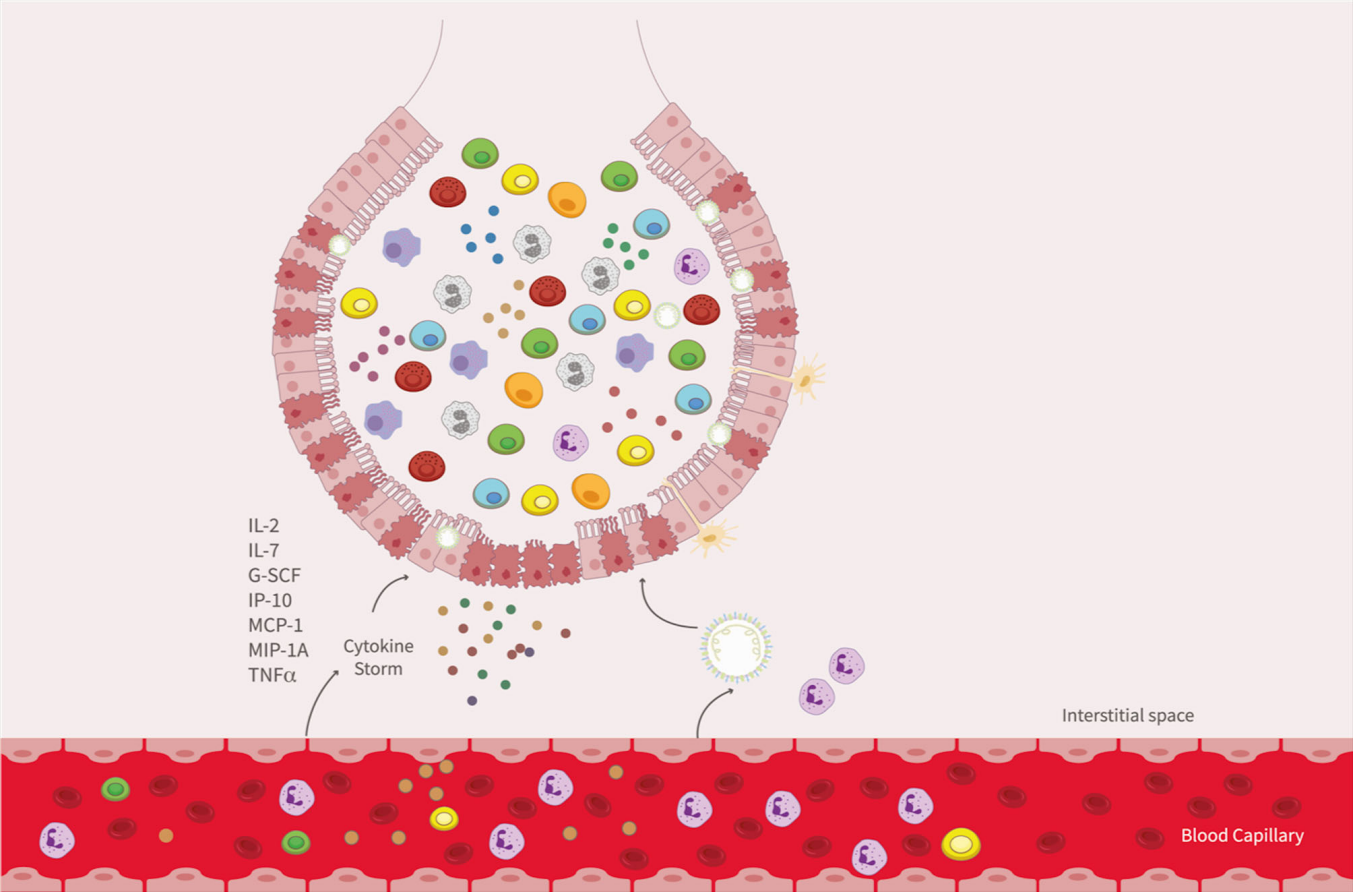
Immune response against SARS-CoV-2. Cellular infiltration and Cytokines storm upon the infection of SARS-CoV-2. After SARS-CoV-2 recognition and replication in type II pneumocytes, peripheral blood cells are recruitment to alveoli, with a release of cytokines and chemokines (IL-2, IL-7, G-SCF, IP-10, MCP-1, MIP-1A, and TNFα), allowing infiltration into the lung of granulocytes and mononuclear cells as monocytes, lymphocytes, and NK cells. Lungs-infiltrated cells are also involving in cytokines storm.

On the other hand, moderate pro-inflammatory cytokines have been observed in mild cases (89, 100), a finding not unique for SARS-CoV-2. Disease severity association with cytokine storm has been broadly studied for viruses such as the respiratory syncytial virus (RSV), which can lead to a cytokine storm syndrome in encephalitis cases (101), influenza A and B virus (102), and others coronaviruses including SARS-CoV (103) and MERS (104), Ebola virus, in hantavirus pulmonary syndrome (105, 106), and Epstein-Barr virus (107). A schematic representation for the contribution of cellular infiltration to disease is shown in **Figure 3**.

### Adaptive Cellular and Humoral Immunity Induced by SARS-CoV-2

Lymphopenia is the most characteristic immune manifestation of patients infected with SARS-CoV-2 (40, 80, 81, 85). Subsets of T cells as CD4^+^, CD8^+^, and memory T cells are lower in severe cases, implying an inadequate antiviral response after SARS-CoV-2 infection (81, 85). In this line, mild cases present an increase of CD8^+^ T cells until 11 days post-admission, which could be associated with the clinical outcome. Noteworthy, a case-report study shows that circulating T follicular helper cells (cTfh) progressively increased after 9 days post-admission during a mild disease and remain in peripheral blood during convalescent, consistent with antibody-secreting cells (ASC) (89). Together with corroborating the data described above, this study also showed the recruitment of ASC and cTfh cells in the blood of patients. These levels are slightly elevated when compared to healthy patients, even after 20 days of symptoms onset (89). Tfh cell function is essential for developing memory B cells and the production of high-affinity antibodies (108). However, more studies are needed to better understand these interactions.

Consistent with this, a higher B/T cell ratio has been found in COVID-19 patients (81, 89). In mild cases, the ASCs are detected after 7 days of post-admission in association with viral clearance (89). Also, in severe cases, ASCs levels are elevated compared to healthy controls, with a 31% expansion 7 days after the onset of the symptoms (109). No significant differences were observed for IgM and IgG production against SARS-CoV-2, with a peak from 7 days until 40 days post-admission independent of clinical course (24, 81, 82, 89, 110). Also, it has been shown that IgG levels in asymptomatic patients were reduced when compared to symptomatic ones, in addition to a reduction in neutralizing antibody activity (24, 81). Along these lines, it was reported that only 19.3% of patients show a robust level of neutralization activity and that they decrease up to 28 days after their recovery, suggesting that neutralizing antibodies have a short half-life (111).

Studies reporting the immunophenotyping of the disease considered innate and adaptative responses, specifically, the neutrophil/lymphocyte ratio (NRL) and the IgG levels. A report, patients were separated into four groups: NRL^high^-IgG^high^; NRL^high^-IgG^low^; NRL^low^-IgG^high^ and NRL^low^-IgG^low^. The worse prognosis was found in both NRL^high^ groups, positively related to fatality. Remarkably, NRL^low^-IgG^low^ patients did not require mechanic ventilation (82). The NRL has been considered a reasonable and helpful measure to define disease severity in patients (112–115). This knowledge has been the key to identifying disease severity markers, which could contribute to defining therapeutic targets and for developing prophylactic strategies and vaccines.

### How the Immune Response Against SARS-CoV-2 Affects the Lung

Due to the broad distribution of ACE2 molecule, SARS-CoV-2 infection could be displaying a multiorgan involvement (116). Nevertheless, the most frequently affected organ is the lung and an acute lung injury (ALI) can develop. Up to date, little is known about the mechanism involved in lung dysfunction. Histopathological analyses from deceased patients have revealed significant changes in the lung, highlighting the alveolar damage with desquamated pneumocytes, hyaline membrane, fibrinous exudate, and immune cell infiltration (117). Specifically, alveolar macrophages are found in the lumen and lymphocytes in the interstitium, associated with diffuse alveolar damage. The high infiltration controls the infection (84, 117–119), consistent with leukopenia in hemogram founded in affected patients. The diffuse damage in the lower respiratory tract could be due to infection in the Clara cells, a non-ciliated secretory type cell, and the evasion of an early immune response, such as interferon type I, allowing a high viral replication in the lower respiratory tract (77, 118, 120).

During the infective process, the virus displays mechanisms to avoid the immune system of the host (78, 79). Once the innate immune cells recognize the viral particles, they become activated with the aim of clear the pathogen. Although peripheral myeloid cells play a crucial role during the innate immune response, little is known about their contribution during SARS-CoV-2 infection. Transcriptomic analyses performed in peripheral blood mononuclear cells (PBMC) and bronchoalveolar lavage fluid (BALF) samples from infected patients revealed a down-regulation of genes related to degranulation and activation of immune cells, such as neutrophils in BALFs (121). Similar results were obtained from studies performed using necropsies (88, 92). On the hand, the expression of pro-inflammatory cytokines and chemokines is upregulated in BALFs of infected individuals (121). Remarkably, genes that encode for chemokine ligands (CCL)-2/MCP-1, CCL-3/MIP-1A, and CCL4/MIP-1β, CXCL2, CXCL8 and CXCL10/IP-10 expression were increased in SARS-CoV-2 patients, suggesting important recruitment of monocytes/macrophages and neutrophils at lung during SARS-CoV-2 infection (121). These findings are consistent with the low levels of peripheral monocytes and lymphocytes that are associated with elevated expression of CCL8 and CCL2 and CXCL9 and CXCL16 in the blood of SARS-CoV-2 patients (77, 122), suggesting the migration of peripheral T cells to control the lung damage from the interstitium to the alveolar space (91). In addition, high levels of CXCL8 were detected in SARS-CoV-2 patients (77), which can promote the recruitment of granulocytes to the tissue and the production a pro-inflammatory environment in the alveoli (123, 124). One explanation for the damage of the airway epithelium is the recruitment of immune cells and the contribution of the degranulation to eliminate the infected cells, promoting an inflammatory state that can last until the viral clearance. As a result of this process, pulmonary damage is produced in a manner equivalent to other respiratory viruses (94, 125, 126).

## Vaccines and Treatments

### Vaccines

Currently, the world science is focused on the development of a vaccine against SARS-CoV-2 (127). The need of developing vaccines during pandemic times represents a major challenge for science and medicine. First, SARS-CoV-2 is a new coronavirus strain, without a complete understanding of the best animal model for this infectious disease. Despite these difficulties, scientists worldwide work to advance with the highest possible velocity to develop and evaluate vaccine prototypes. Inactivated virus, DNA-based strategies, non-replicating viral vector, protein subunits, replicating viral vector, live attenuated virus, RNA-based, and VLPs are within the strategies being developed (128). In the last WHO report, 152 vaccines prototypes are being tested in preclinical models, and 42 are tested in humans, with ten of them in phase (129). Besides, until October 15, 2020, 10 studies, which showed immunogenicity and safety in previous phases, are in the early stages of Clinical Phase 3 (**Table 1**). As part of these phase 3 studies, between 30,000 and 65,000 volunteers have been recruited to evaluate these vaccine prototypes around the world (130).

**Table 1:** Vaccine candidates for COVID-19 currently in clinical trials and their strategies.

	Vaccine candidates	Phase I	Phase I/II	Phase II	Phase III
**Non-Replicating Viral Vector**	ChAdOx1-S		PACTR202006922165132	2020-001228-32	ISRCTN89951424 NCT04516746
	Adenovirus Type 5 Vector	ChiCTR2000030906		ChiCTR2000031781	NCT04526990
	Ad26COVS1		NCT04436276		NCT04505722
	Adeno-based (rAd26-S+rAd5-S)	NCT04436471 NCT04437875			NCT04530396
	Replication defective Simian Adenovirus (GRAd) encoding S	NCT04528641			
**Inactivated**	CoronaVac		NCT04383574 NCT04352608		NCT04456595669/UN6.KEP/EC/2020
	Inactivated		ChiCTR2000031809		ChiCTR2000034780
	Inactivated		ChiCTR2000032459		ChiCTR2000034780
	Inactivated	NCT04412538	NCT04470609		
	Inactivated		NCT04530357		
	Whole-Virion Inactivated		NCT04471519		
**Protein Subunit**	Adjuvanted recombinant protein (RBD-Dimer)	NCT04445194		NCT04466085	
	Full length recombinant SARS CoV-2 glycoprotein nanoparticle vaccine adjuvanted with Matrix M		NCT04368988		
	RBD-based		NCT04473690		
	Native like Trimeric subunit Spike Protein vaccine	NCT04405908			
	Recombinant spike protein with Advax™ adjuvant	NCT04453852			
	Molecular clamp stabilized Spike protein with MF59 adjuvant	ACTRN12620000674932p			
	S-2P protein + CpG 1018	NCT04487210			
	RBD + Adjuvant	IFV/COR/04			
	RBD (baculovirus production expressed in Sf9 cells)	ChiCTR2000037518			
	Peptide (EpiVacCorona)	NCT04527575			
**Virus like-particles**	Plant-derived VLP adjuvanted with GSK or Dynavax adjs.	NCT04450004			
**RNA**	LNP-encapsulated mRNA	NCT04283461		NCT04405076	NCT04470427
	3 LNP-mRNAs		2020-001038-36 ChiCTR2000034825		NCT04368728
	mRNA	NCT04449276		NCT04515147	
	mRNA		NCT04480957		
	LNP-nCoVsaRNA	ISRCTN17072692			
	mRNA	ChiCTR2000034112			
**DNA**	DNA plasmid vaccine with electroporation		NCT04447781 NCT04336410		
	DNA plasmid vaccine + Adjuvant		NCT04463472 NCT04527081		
	DNA plasmid vaccine		CTRI/2020/07/026352		
	DNA Vaccine (GX-19)		NCT04445389		
**Replicating Viral Vector**	Measles-vector based	NCT04497298			

The inactivated virus formulation has been broadly used for licensed vaccines being used for decades to prevent emerging respiratory diseases (131). Phase 1/2 clinical trials revealed the safety of using an inactivated vaccine against SARS-CoV-2 with a range of adverse reactions between 20% and 30% of those vaccinated and a robust neutralizing response after the second dose (132–134).

An inactivated vaccine generated by Sinovac (CoronaVac) was evaluated pre-clinically rats, mice and rhesus macaques, with two doses, on days 0 and 7 days after challenge, secretion of IgG of 10^5^ approximately 1 week after immunization against the SARS-CoV-2 S-protein important to obtain a robust IgG secretion. Consistently, neutralization assays were carried out. A high titer of neutralized antibodies (1 x 10^4^ approximately) is observed 6 weeks after immunization, the results are similar for clinical phase 1 and 2 study with two doses at 0 and 14 days (133, 134). Also, Coronavac has not shown significant severe adverse reactions in Phases 1 and 2. Thus, this vaccine has moved on to a Phase 3 clinical trial (135).

The inactivated vaccine developed by Sinopharm evaluated in Phases 1 and 2 clinical trials was administrated intramuscularly in two doses (5 ug/dose) at 0 and 21 days. According their interim reported showed high titers of neutralized antibodies in volunteers, 1 x 10 ^3^ approximately (132). Currently, this vaccine is in Phase 3 (135).

BBIBP-CorV is an inactivated prototype of a vaccine against SARS-CoV-2 tested in six clinical models (including mice, rats, guinea pigs, rabbits, and nonhuman primates), which were immunized intramuscularly route twice on days 0 and 21. These mammalian species were challenged with SARS-CoV-2; the vaccine-induced high levels of neutralizing antibodies do not produce significant adverse effects in the biochemical serum parameters. Furthermore, it was observed that immunization with two doses with 2 ug/dose of BBIBP-CorV conferred significant protection against SARS-CoV-2 (136).

Adenovirus-based vaccines are led by AstraZeneca, CanSino Biologics Inc, Gamaleya Research Institute, and Janssen Vaccines & Prevention B.V (130). AstraZeneca produced a chimpanzee adenovirus-vectored vaccine (ChAdOx1 nCoV-19) in their report indicated the vaccine was tolerated with 1 g of paracetamol every 6 h, which must be administered prophylactically 24 h before vaccination (137). The response of IgG antibodies against the SARS-CoV-2 protein S reached a peak on day 28 and was maintained until day 56 after vaccination. Furthermore, the IFN-γ response peaked at day 14 and antibodies capable of neutralizing live SARS-CoV-2 were induced on day 28 after a booster dose (137). The Phase 3 clinical trial of this vaccine prototype was paused to allow the review of data by an independent committee, due to a suspected severe adverse reaction in two different patients. After this review, the trial was restarted in India, Brazil, and the UK (138).

CanSino Biologics INC developed a prototype with recombinant adenovirus type 5 (Ad5) called rAd26-S + rAd5-S, which expresses the S glycoprotein of SARS-CoV-2. In phase 1/2 studies it was shown that the vaccine is safe, well-tolerated, with the majority of adverse reactions of mild or moderate severity which include fever (46%), headache (39%), fatigue (44%), vomiting (2%), loss of appetite (16%), diarrhea (11%), and occur in the first 7 days post-vaccination. In general, this vaccine induced a strong humoral and cellular immune response in healthy patients, which begins to be observed 14 days post-vaccination. Secretion of IFN-γ, TNF-α, and IL-2 by CD4^+^ and CD8^+^ T cells was measured, and a peak was observed at day 28th post-vaccination). A single dose of the COVID-19 vaccine vectorized with Ad5 caused a four-fold increase in RBD-binding antibodies and virus neutralization (139). Due to the success of this prototype, it had advanced to a Phase 3 clinical study (129).

A preliminary report from a phase 1 for a mRNA-based vaccine against SARS-COV-2 (mRNA-1273) indicated that seroconversion appears on day 15 after the first vaccination (140). Also, antibody neutralizing activity was detected after the second vaccination (140). A report of phase 1/2 of RNA vaccine BNT162b1 indicated that the candidate elicited a higher titer of nAb than the convalescent group, with a temporal decrease of lymphocytes that return to baseline levels between 1 week (141). No data for T and B cell immunity have been reported yet (141).

Subunit vaccines used protein-S, and RBD subunits since S protein must have a stable domain (142, 143). It was shown that spike-immunized mice developed a virus neutralizing antibody response that lasted for 10 weeks approximately (144). Novavax designed a prototype with an innovative adjuvant Matrix-M1 adjuvant, a positive correlation between the immunization dose mixed with adjuvant with the production of neutralized antibodies was obtained (145). Also, this prototype is evaluated in Phase 3 (138).

The recombinant viral vector methodology has been widely studied for the development of vaccines and gene therapy (146). Viral vectors used for this strategy are measles virus, poxvirus, adenovirus, and vesicular stomatitis virus (147). This type of prototype has been widely studied with adenoviruses since they can induce a population of CD4^+^ and CD8^+^ cells (148, 149). For SARS-CoV-2, this vaccination strategy has used the vesicular stomatitis virus (VSV) with replicative capacity, which expressed a modified form of the S-gene of SARS-CoV-2. This preliminary study demonstrated a decrease in viral load in mice immunized with two doses of VSV-eGFP-SARS-CoV-2 and challenged with SARS-CoV-2, at 4 days post-infection in samples of lung, splenocytes, heart, and nasal lavage, in addition to high IgG secretion (150).

### Trained Immunity and SARS-CoV-2 Infection

Trained immunity is based on acquiring a type of immune memory by epigenetic reprogramming of innate immune cells, such as monocytes and NK cells (151, 152). It has been observed that the tuberculosis vaccine consisting of an attenuated mycobacteria Bacillus Calmette-Guerin (BCG) produces a long-lasting trained immunity consisting of these innate cell populations (153–157). It has been hypothesized that BCG-vaccination could associate with decreased disease severity and lethality during COVID-19 worldwide (158). The lethality reported by COVID-19 in countries with BCG vaccination is about 5.8 times lower than countries without the BCG vaccination program (159). Equivalent suggestions have been made by other studies supporting the notion of using the BCG vaccine to prevent severe COVID-19 at least in the most vulnerable groups, such as the elderly and healthcare personnel (160, 161). Recently, a study in a hospital from the United Arab Emirates demonstrated that a boost with the BCG vaccine in the healthcare workers could reduce the disease caused by SARS-CoV-2 (162).

However, clinical trials are needed to scientifically define the impact of BCG vaccine as a preventive measure for COVID-19. Along these lines, since WHO declared a pandemic for SARS-CoV-2, more than 21 clinical trials are currently in progress to evaluate whether BCG vaccination in adults and healthcare workers could decrease the symptoms caused by COVID-19 (163). Some of these first clinical trials were performed in the Netherlands (NCT04328441) and Australia (NCT04327206) as phase III clinical trials (163). In addition, one phase IV clinical trial is in progress (NCT04417335).

Despite the promising results of BCG vaccination, up to date there are not yet published data demonstrating the benefits of the BCG vaccine in clinical trials. Moreover, the WHO did not recommend the BCG vaccine as an approach for COVID-19 (164). It is likely that the WHO decision was based on the lack of direct clinical evidence supporting a benefit for SARS-CoV-2 infection and that an increased demand for the BCG vaccine could jeopardize the supply for tuberculosis rates vaccination (164).

### Therapeutic Approaches for SARS-CoV-2 Infection

The design of therapeutic strategies for COVID-19 has been oriented in three fundamental directions: viral elimination using molecules that can interfere with viral replication, anti-inflammatory therapies, or palliative treatments that help reduce symptoms and the regulation of the immune response (165). The COVID-19 emergency had challenged researchers to design treatments. In the meantime, clinicians have given some already registered drugs to reduce disease severity in patients.

Furthermore, recent findings suggest that clinical recovery can be promoted by the administration of antiviral therapy before lung disease is at an advanced stage (166, 167). In this section, some drugs and other therapeutic strategies under evaluation will be described. A summary of the therapeutic approaches is described in **Table 2**.

**Table 2:** Treatments under evaluation for COVID-19 and their mechanisms of action.

	Treatments	Action Mechanisms	Limitations
**Antivirals**	*Remdesivir*	Viral replication inhibitor (168)	Bradycardia, liver damage, and gastrointestinal reactions that can contribute to the deterioration of the disease (169).
	*Favipiravir*	Inhibits viral RNA polymerase (170).	Gastrointestinal disorders, skin lesions, liver, and cardiovascular damage (168, 171).
	*Lopinavir-ritonavir*	Protease inhibitor believed to interfere with viral fusion with cell membrane (172, 173).	Antiviral activity against SARS-CoV-2 has not been found in clinical studies (174, 175).
	*Arbidol*	Blocks entry and intracellular traffic in vesicles (176).	Some patients may develop hypersensitivity (176).
**Other drugs**	*Chloroquine and hydroxychloroquine*	Immunomodulatory effect. It is inhibiting the entry of SARS-CoV-2 (168, 177–179).	Accumulation in various organs and low elimination rate; and arrhythmia and heart failure (178, 180, 181).
	*Melatonin*	Inhibits calmodulin (182, 183).	This would only allow reducing the associated clinical symptoms (183).
	*Dexamethasone*	Reduce the duration of mechanical ventilation and mortality from severe acute respiratory distress syndrome (184).	There are only a palliative treatment (184).
	*Plasma Treatment*	Neutralizing antibodies against SARS-CoV-2 (185).	Doses dependent on the neutralizing title (186).
	*Specific monoclonal antibodies to SARS-CoV-2*	Blocks the entry of the virus through ACE2 because they are mainly directed against the S protein (187–190)	Although several mAbs have been evaluated *in vitro*, *in vivo*, and there are ten clinical trials ongoing (166, 170, 171, 191)
	*Tocilizumab*	Non-specific Abs for SARS-CoV-2. This directed against IL-6 receptor, IL-6 is one of the cytokines responsible for the inflammatory state persistent in severe patients (192, 193).	It has been reported only beneficial effects in critically ill patients (82, 193).

#### A) Antivirals

Remdesivir is a nucleotide analog that works as an inhibitor upon binding to the viral RNA strands and disrupts new the synthesis viral genomes with successful results against coronaviruses as SARS-CoV MERS-CoV, is currently tested for SAR-CoV-2 (168, 194). *In vitro* studies in Vero E6 cells have shown that Remdesivir inhibits the replication of SARS-CoV-2 after entry into the cells (168). Medical reports published by China and the United States indicate a faster recovery of patients in response to the administration of Remdesivir (92, 169). Recently, the Food and Drug Administration (FDA) authorized this drug for severely hospitalized COVID-19 patients (195). Later, the FDA approved the use of Remdesivir in hospitalized patients with confirmed or suspected COVID-19, regardless of severity, based on the results in a controlled clinical trial including hospitalized patients with mild, moderate, and severe disease (NCT04280705).

Favipiravir is a purine nucleic acid analog previously used to treat influenza, considered a prodrug whose active metabolite interferes with viral replication by inhibiting viral RNA polymerase (170, 191). In a clinical trial (ChiCTR2000029600), the administration of Favipiravir combined with interferon−α (IFN−α) in patients with COVID-19, contributes to an enhanced viral clearance (171).

Lopinavir and ritonavir are protease inhibitors used in the therapy for human immunodeficiency virus 1.(HIV-1) (196). SARS-CoV-2 patients treated with these drugs showed only a slight improvement (4, 5, 197). The combination of lopinavir-ritonavir with ribavirin and IFNβ/1b reduced viral loads in nasopharyngeal swabs, stool, and saliva obtained from patients after 8 days of treatment (198). Notably, IL-6 levels decreased significantly after treatment with this combined antiviral treatment (174). These observations suggest that an early treatment with these drugs can decrease virus spread and reduce the exacerbated production of pro-inflammatory cytokines (198).

Arbidol is a derivative of indole, which acts on aromatic amino acids and can interfere with the replication of the influenza virus (199). *In vitro* studies on SARS-CoV-2 have shown that Arbidol can block the entry and trafficking to intracellular vesicles (176). Arbidol reduces the risk of developing lung lesions when used alone or together with lopinavir/ritonavir to promote viral elimination without significant side effects (200–202). Additionally, the mix of antivirals and interferon was shown to efficiently reduce both symptoms and viral loads (203). Currently, a clinical study is evaluating the safety and efficacy of Arbidol as an adjuvant for an antiviral therapy combined with IFNβ/1a in SARS-CoV-2 positive patients (NCT04350684).

The main restriction of antiviral treatments is the development of adverse effects, such as liver and cardiovascular damage, gastrointestinal disorders, and skin lesions, which are the most commonly reported for the antivirals mentioned above and could worsen the patient condition (168, 169, 171).

#### B) Aminoquinolines

Chloroquine and its hydroxychloroquine analog are antimalarials that have been used for over 50 years as a prophylaxis and treatment (204). Both drugs lead to a reduction of MHC class II expression, the production of reactive oxygen species (ROS) and pro-inflammatory cytokines, such as IL-1β and TNF-α (204). Additionally, hydroxychloroquine can enter lysosomes, impairing the acidification mechanisms and the function of these organelles (205).

Previous data from *in vitro* studies showed that chloroquine can display an antiviral capacity against Chikungunya virus (CHIK) and SARS-CoV (177, 206). Furthermore, chloroquine was shown to be able to inhibit the entry of SARS-CoV-2 into Vero E6 cells at concentrations that do not cause cytotoxic effects *in vivo* (168). Due to these results, both drugs were applied as treatment to COVID-19 patients worldwide, with a constant debate about effectiveness (207). However, later on during the pandemic, WHO suspended all clinical trials testing these compounds for COVID-19, as no significant improvements were found in treated patients. Furthermore, one clinical study showed a high fatality rate (63.6%) due to high hydroxychloroquine doses, which can cause cardiac toxicity (180, 181, 208).

#### C) Melatonin

N-acetyl-5-methoxytryptamine (or melatonin) is a derivative of tryptophan, an essential amino acid that plays a critical role in immuno-inflammatory events produced during a viral infection. Besides, melatonin acts as an antioxidant that reduces oxidative stress and reduces vascular permeability (209–212). The use of this molecule use has increased in atherosclerosis and respiratory distress, such as acute lung disease, viral or bacterial infections (209–212). Melatonin alleviates respiratory pain by reducing vascular permeability and can inhibit calmodulin, an essential protein for ACE2 function (182). Because melatonin indirectly targets various cellular targets of SARS-CoV-2, including the ACE2, the use of this molecule against SARS-CoV-2 infection has been suggested (183, 213). Currently, six clinical studies are evaluating the effectiveness and safety of melatonin as prophylaxis, both in critically ill and outpatients (NCT04474483, NCT04531748, NCT04409522, NCT04353128, NCT04530539, NCT04470297). Two of these clinical studies are currently in the recruitment phase, the first plans to evaluate the anti-inflammatory effects of melatonin in a patient with COVID-19 (NCT04409522). The second takes advantage of the anti-inflammatory effects of melatonin to proposed it as prophylaxis for health care workers (NCT04353128). However, it must be considered that this drug does not inhibit viral replication and transcription. Instead, the administration of melatonin only reduces the associated clinical symptoms (182, 214).

#### D) Dexamethasone

Dexamethasone is a corticosteroid that has been shown to reduce the duration of mechanical ventilation and fatality caused by COVID-19 (215). The use of corticosteroid for the management for acute respiratory distress syndrome due to viral infections is a controversial topic since these drugs can delay the elimination of the virus and increase the risk of secondary infections due to their immunosuppressive effect. Despite this, it has been used successfully to treat influenza A (H1N1) pdm09-associated pneumonia, reducing mortality (216). Thus, dexamethasone can be used as a treatment combined with other antiviral or drug therapy (186, 215, 217).

#### E) Immune Therapies

##### i) Plasma Transfusion

Transfusion of plasma is used for acute infections and is a classic form of immunotherapy for emerging infectious-diseases (218). This therapy is based on the passive administration of plasma with high titers of nAbs, generated endogenously by convalescent individuals (219). Such a therapy is still in use and has been implemented successfully in diseases without a favorable treatment or specific vaccine (187). During the last epidemics due to Ebola (EBOV), Influenza A-H1N1, and emerging coronaviruses, this type therapy has been implemented as an alternative for treatment (220–223).

Mainly, transfer of plasma from convalescent patients after SARS-CoV or MERS infections showed viral loads reduction and improvement of clinical and laboratory parameters, with a decrease in temperature, increased oxyhemoglobin saturation, improved lymphocyte levels, and decreased C-reactive protein levels. Also, fresh frozen plasma (FFP) improves survival rates in patients (186, 224). Similarly, recovery from COVID-19 disease was observed in patients with severe illness after treatment with convalescent plasma with high nAbs titters obtained from surviving donors (185). Importantly, during an FFP treatment, other biological products involved in the recovery of patients, such as anti-inflammatory cytokines and defensins, are also transferred (225). The principal limitation of FFP is to determine the optimal nAb titer required for an efficient virus neutralization (186). Thus, hemovigilance is essential because some patients could need more than one dose of FFP depending on the severity of illness, as described for other viral diseases where plasma transfusion was utilized (226). Moreover, the use of FFP has equivalent risks for adverse effects than any other blood component transfusion, such as allergic/anaphylactic reaction and infections due of other microbes (226).

Nevertheless, the reported incidence of adverse reactions after transfusion of convalescent plasma is less than 5% (227). One of the precautions to consider is avoiding Transfusion Related Acute Lung Injury (TRALI) (218, 227). TRALI is a new acute lung injury and occurs during or within 6 h after transfusion (218, 228). TRALI-patients present acute dyspnea, need of intubation, hypertension, hypotension, and acute leukopenia (218, 228). To prevent acute lung injury, it has been recommended to transfer the plasma to patients who have never been pregnant or undergone an abortion, thus allowing to decrease the possibility of presenting the antibodies to HLA or granulocyte antigens in the serum that could lead to the development of TRALI (218, 229).

The half-life of antibodies determinates the election for the plasma donor (226). Yet, researchers have not determined the reduction of viral loads in severe COVID-19 patients with convalescent plasma treatment (CPT) or FFP and mixture with antiviral treatments (187, 224, 230). Furthermore, given that the population at risk includes patients with comorbidity such as hypertension, diabetes, or cardiovascular diseases, which are part of potential CPT donors, makes it challenging to validate CPT as one of the therapies that should be considered on a larger scale (224, 230, 231).

Despite all these questions, both advantages and disadvantages of the therapy should be taken into consideration for treatment of SARS-CoV-2 infection. Based on history and evidence, and due to the health emergency, CTP is currently used throughout the world and was recently approved by the FDA, with guidelines for healthcare professionals (232, 233).

##### ii) Monoclonal Antibodies

Monoclonal antibodies (mAbs) are a powerful tool for treating of several diseases, such as cancer and immunological illnesses (234). Along these lines, various strategies are in progress to isolate different mAbs specific for various SARS-CoV-2 antigens, due the potential of mAbs against emerging viruses (192, 235). Further, the use of mAbs against other emerging viruses has demonstrated potent neutralizing effects for SARS-CoV and MERS-CoV (234, 236). Since mAbs have been previously tested as prophylactics for SARS, using mAb to prevent SARS-CoV-2 infection stands as a promising approach to treat or prevent this disease, at least in the most susceptible individuals (235, 237). Additionally, mAbs could be used as treatment against viral encephalitis, and their use might help reduce not only respiratory symptoms by also neurological symptoms (238). For this reason, several groups are working on the development of this therapy for COVID-19 using various strategies.

Human recombinant soluble ACE2 (hrsACE2) blocks the viral entry *in vitro*, decreasing viral loads, however, *in vivo*, hrsACE2 failed to block viral entry (192). Other strategies involve the development of mAbs from SARS-CoV-2 convalescent individuals and 206 mAbs were recently isolated from eight of them. Although two of those antibodies blocked viral entry, their specificity and neutralizing activity requires further characterization (239). More recently, a new mAb was discovered called MAb362, which is a cross-reactive human IgA mAb that was shown capable to neutralize SARS-CoV-2 *in vitro*. This mAb also targets the S protein and prevents the binding with the ACE2 receptor (235).

Similarly, nineteen nAbs that target the RBD or the N-terminal domain (NTD) from the S protein were isolated from convalescent patients (240). All the antibodies demonstrated an impressive capability of neutralizing SARS-CoV-2 *in vitro*, making them good candidates for clinical evaluation (240). Additionally, the isolation of neutralizing mAbs against SARS-CoV-2 from convalescent patients of COVID-19 led to the identification of two mAbs, CA1, and CB6. While both showed *in vitro* the capacity to neutralize the virus, CB6 outperformed CA1. Studies in the rhesus macaque model showed decreased viral loads due to intravenous vaccination at a dose of 50 mg/kg with CB6. Furthermore, a single dose of CB6 prophylaxis before exposure to SARS-CoV-2 demonstrated protection in macaques against infection (241).

REGN-COV2 is an anti-viral antibody cocktail, which is composed by anti-spike antibodies that target the RBD of the protein (242). This cocktail has the potential of being used as prevention as well as a treatment for patients with COVID-19 (242, 243). Currently, REGN-COV2 is being evaluated in clinical trials as a treatment for non-hospitalized and hospitalized COVID-19 patients, and as prevention for the high-risk group of people (243).

Other mAbs have been tested in patients with COVID-19, such as tocilizumab, an IL-6 receptor-specific mAb that was evaluated in at least one clinical trial (ChiCTR2000029765) showing beneficial effects in a cohort with severe COVID-19. Also, these mAbs were recommended for patients with low IgG levels and high NLR (82). Nonetheless, adverse effects reported included increased hepatic enzymes (transaminases), thrombocytopenia, neutropenia, cutaneous rash and infections in the bloodstream. Other side effect were bacteremia and fungemia, which were likely the result of the IL-6 inhibition, which can impair B and T cell proliferation and function and diminish the immune response against infection agents (244). Furthermore, mAbs capable of neutralizing pro-inflammatory cytokines could be applied to prevent or reduce the cytokine storm (82, 193).

Few studies are ongoing to identify and obtain mAbs against SARS-CoV-2, such as the NCT04342195 and NCT04354766. More than ten clinical trials evaluating the effectiveness of mAbs as a treatment against this virus are in progress (245). Within the first trials are the involved the use of TJ003234 (NCT04341116), gimsilumab (NCT04351243), and lenzilumab (NCT04351152), which are mAbs against granulocyte macrophage-colony stimulating factor (GM-CSF). The last one is currently in phase III clinical trial (245). Other phase III trials in progress are REGN-COV-2 (NCT04452318) that has three different targets in the S protein, thus preventing the virus from entering into the cell, and LY-CoV555 (NCT04497987) that comes from a patient now recovered from SARS-CoV-2 (246). Although no results have been published up to date, these mAbs seem as promising approaches for preventing or treating COVID-19.

##### iii) Single-Domain Antibodies

Heavy chain antibodies are derived naturally from camelids (247, 248). Their lack of variable region has a smaller size than conventional antibodies, with high stability and affinity for each respective cognate epitope (247, 248). Therefore, the isolation of a camelid-derived single domain antibody (SdAb) is considered a new promising therapeutic strategy (248). Similar to mAbs, the therapeutic mechanism is focused on the virus neutralizing activity. For this reason, SARS-CoV-2 is mainly directed against RBD, blocking the union with the ACE2 receptor. A SdAb called H11 was identified with a higher affinity, neutralizing activity and a K_D_ <1 μM (249). Similarly, a SdAb called Ty1 was isolated from an alpaca after immunization twice with SARS-CoV-2 S1-sheep-Fc and the other two with SARS-CoV-2 RBD region in a 60 days scheme (247).

## Concluding Remarks

Even though it has been more than 6 months since discovering this novel coronavirus, the knowledge of the immune response elicited against SARS-CoV-2 remains scarce. So far, it has been proposed that the immune response is a major component of the pathology observed during COVID-19.

The reduction of T cells in peripheral blood during SARS-CoV-2 infection could lead to an inadequate memory response, similar to what is described for other viral infections (81). It has been proposed that a limited presence of eosinophils, monocytes, and T cell subsets in peripheral blood might be due to the large recruitment of those cells to the lung epithelium after infection (**Figure 3**) (92). Consistently with this notion, it was shown that SARS-CoV and MERS impaired the host memory immune response (250). Although, the long-lasting immune response cannot be elucidated yet, a vaccine for SARS-CoV-2 is expected to promote long lasting immunological memory (251–253).

Furthermore, there are still many unknown elements of the immune response induced by SARS-CoV-2. The severe clinical cases have allowed us to begin understanding the development of SARS-CoV-2 infection in the host. Despite this, many questions have arisen about the immunopathology and as to how it can relate to pre-existing chronic diseases and severe clinical manifestations, even death in young people. Up to date, the number of lymphocytes and neutrophils have been suggested as markers of disease severity (254). As described above, several studies have revealed symptoms involving the CNS during COVID-19 disease. However, it has not yet been defined the mechanism for viral entrance in CNS. Additional studies are needed to explain as to how cytokine storm is associated to disease severity and patient with the outcome.

Scientific efforts to design therapies are essential to prevent acute respiratory deterioration, the use of ventilation, morbidity, and mortality from SARS-CoV-2 infection. Although during the pandemic, significant progress on vaccine development has been made, still no safe and efficacious vaccine is available for the use of the population. The fact that several of prototypes are currently starting phase III clinical trial evaluation (**Table 1**) provides hope for an availability of vaccine for COVID-19 much sooner than for any other infectious disease. Furthermore, production of mAbs could be a parallel strategy for COVID-19 prophylaxis.

It seems likely that an unbalanced immune response triggered by SARS-CoV-2 infection requires that the therapeutic interventions described above not only aim at eliminating the virus but also at regulating the immune response to prevent the exacerbated inflammation observed during COVID-19 (255). The antivirals target different steps of viral replication, the combination of which could be useful to limiting viral loads and the subsequent spread of the virus, promoting an effective antiviral response by T cells (10, 11). The combination of antiviral therapies with interferon can be beneficial to control viral spread (256). In addition, some antiviral drugs are capable of activating a cellular immune response on its own, stimulating the secretion of virus-specific antibodies and activating adaptive immunity (170, 257). Finally, blocking the pro-inflammatory cytokine response is another crucial point for the design of therapeutic strategies to improve critically ill patients (82).

The search for a vaccine is an urgent need at a global level. Nowadays, technologies have allowed us to work by leaps and bounds searching for new vaccines or therapies against SARS-CoV-2. It is necessary to prioritize the health of volunteers and ensure the safety of vaccine prototypes, especially since the variety of potential adverse effects have not been fully understood yet, such as the antibody-dependent enhancement (ADE), which stands as a risk of vaccination or immunotherapy. A full study regarding all the possible adverse effect is needed, as well as the transparency of the clinical studies that are carried out.

## Author Contributions

GC-M designed, wrote, and revised the manuscript, and designed the figures. FS wrote, revised, and edited the manuscript. CA wrote, revised, and edited the manuscript. MO and CR wrote and revised the manuscript. LR-G and RB wrote and drew the figures. AK is the leading investigator and supported in the organization and full manuscript revision. All authors contributed to the article and approved the submitted version.

## Funding

This work was supported by FONDECYT 1190830, FONDEF D11I1080, Millennium Institute on Immunology and Immunotherapy (P09/016-F, ICN09_016), Fundación COPEC-UC, Regional Government of Antofagasta through the Innovation Fund for Competitiveness FIC- R 2017 (BIP Code: 30488811-0). Biomedical Research Consortium Chile (13CTI-21526/P4). AK is a Helen C. Levitt visiting professor at the Department of Microbiology and Immunology of the University of Iowa.

## Conflict of Interest

The authors declare that the research was conducted in the absence of any commercial or financial relationships that could be construed as a potential conflict of interest.
